# Biochemical Characterization of New Gemifloxacin Schiff Base (GMFX-o-phdn) Metal Complexes and Evaluation of Their Antimicrobial Activity against Some Phyto- or Human Pathogens

**DOI:** 10.3390/ijms23042110

**Published:** 2022-02-14

**Authors:** Hazem S. Elshafie, Sadeek A. Sadeek, Ippolito Camele, Amira A. Mohamed

**Affiliations:** 1 School of Agricultural, Forestry, Food and Environmental Sciences, University of Basilicata, 85100 Potenza, Italy; hs.elshafie@gmail.com; 2 Department of Chemistry, Faculty of Science, Zagazig University, Zagazig 44519, Egypt; s_sadeek@zu.edu.eg; 3 Department of Basic Science, Zagazig Higher Institute of Engineering and Technology, Zagazig 44519, Egypt; amira222283@yahoo.com

**Keywords:** biological ligand, metal complexes, chelation theory, phytopathogens, human pathogens, antioxidants

## Abstract

Four novel ligand-metal complexes were synthesized through the reaction of Fe(III), pleaseCo(II), Zn(II), and Zr(IV) with Schiff base gemifloxacin reacted with ortho-phenylenediamine (GMFX-o-phdn) to investigate their biological activities. Elemental analysis, FT-IR, ^1^H NMR, UV-visible, molar conductance, melting points, magnetic susceptibility, and thermal analyses have been carried out for insuring the chelation process. The antimicrobial activity was carried out against *Monilinia fructicola*, *Aspergillus flavus*, *Penicillium italicum*, *Botrytis cinerea*, *Escherichia coli*, *Bacillus cereus*, *Pseudomonas fluorescens,* and *P. aeruginosa*. The radical scavenging activity (RSA%) was in vitro evaluated using ABTS method. FT-IR spectra indicated that GMFX-o-phdn chelated with metal ions as a tetradentate through oxygen of carboxylate group and nitrogen of azomethine group. The data of infrared, ^1^H NMR, and molar conductivity indicate that GMFX–o-phdn reacted as neutral tetra dentate ligand (N_2_O_2_) with metal ions through the two oxygen atoms of the carboxylic group (oxygen containing negative charge) and two nitrogen atoms of azomethine group (each nitrogen containing a lone pair of electrons) (the absent of peak corresponding to ν(COOH) at 1715 cm^−1^, the shift of azomethine group peak from 1633 cm^−1^ to around 1570 cm^−1^, the signal at 11 ppm of COOH and the presence of the chloride ions outside the complex sphere). Thermal analyses (TG-DTG/DTA) exhibited that the decaying of the metal complexes exists in three steps with the final residue metal oxide. The obtained data from DTA curves reflect that the degradation processes were exothermic or endothermic. Results showed that some of the studied complexes exhibited promising antifungal activity against most of the tested fungal pathogens, whereas they showed higher antibacterial activity against *E. coli* and *B. cereus* and low activity against *P. fluorescens* and *P. aeruginosa*. In addition, GMFX-o-phdn and its metal complexes showed strong antioxidant effect. In particular, the parent ligand and Fe(III) complex showed greater antioxidant capacity at low tested concentrations than that of other metal complexes where their IC_50_ were 169.7 and 164.6 µg/mL, respectively.

## 1. Introduction

The crystal structures of several free fluoroquinolones molecules have been determined in several research [1,2,3]. It is interesting to note that in most cases the carboxylic group is not deprotonated and the hydrogen atom of this group is hydrogen bonded to an adjacent 4-oxo atom. In few examples such as gemifloxacin, lomefloxacin, norfloxacin, moxifloxacin, and ciprofloxacin, the carboxylic group is protonated and the molecules exist in a zwitterionic form with protonated terminal nitrogen of the piperazine ring in a solid state [4,5,6]. Crystal structures of fluoroquinolones complexes indicate that neutral fluoroquinolones in the zwitterionic state are capable of forming simple complexes bidentately through one of the carboxylic oxygen and ring carbonyl oxygen [7,8,9,10].

Gemifloxacin is the fourth generation of quinolones (or quinolonecarboxylic acids) that are an essential group of antibacterial agents containing 4-oxo-1,4-dihydroquinoline skeletons and are widely utilized in the medicament of many infections [11,12,13]. The addendum of the fluorine atom to the main quinolones produces fluoroquinolones, which have a much wider range of activity and improved pharmacokinetics [14,15,16]. Gemifloxacin is also an antibacterial drug with superior in vitro activity versus both Gram-positive (G + ve) and Gram-negative (G − ve) bacteria [17,18]. Gemifloxacin is also reported to have an anticancer effect on colon cancer through metastasis inhibition [19]. It should be noted that gemifloxacin inhibits the action of DNA gyrase and topoisomerase IV, thus inhibiting DNA replication and eventually bacterial growth [20,21]. Gemifloxacin reacted with primary or secondary amine forming Schiff base [22,23,24]. Schiff bases are well-known to coordinate with most transition metal ions as a bidentate or tridentate, and their complexes are recorded [25]. In many areas, such as industrial, agricultural, and pharmaceutical chemistry, Schiff bases and their metal complexes have found vast applications [26,27,28,29]. In vision of the above consideration the current paper deals with the reaction of some elements such as Fe(III), Co(II), Zn(II), and Zr(IV) on the efficiency of gemifloxacin in the new form GMFX-o-phdn benzene-1,2-diamine [7 -[(4Z)-3(aminomethyl)-4-(methoxyimino) pyrrol dine-1-yl]-1-cyclopropyl-6-fluoro-4-oxo-1,8-naphthyridine-3-carboxylic acid]. The solid chelates were characterized using different physio-chemical techniques like elemental analyses (C, H, and N), IR, UV-Visible, ^1^H NMR, molar conductance, magnetic susceptibility, melting points, thermo gravimetric (TG/DTG), and differential thermal (DTA) analyses. Moreover, the kinetic and thermodynamic parameters for the different thermal degradation steps of the compounds were determined by Coats–Redfern and Horowitz–Metzger methods. The antifungal activity was evaluated against four phytopathogenic fungi: *Monilinia fructicola* (G. Winter) Honey, *Aspergillus flavus* Link, *Penicillium italicum* Wehmer, and *Botrytis cinerea* (de Bary) Whetzel. Whereas the antibacterial activity was evaluated against *Escherichia coli* Migula, *Bacillus cereus* Frankland & Frankland, *Pseudomonas fluorescens* Flügge (Migula), and *P. aeruginosa* Schroter. Furthermore, the antiradical activity of the studied compounds was evaluated using 2,2′-azinobis (3-ethylbenzthiazoline-6-acid) (ABTS•+) assay.

## 2. Results and Discussion

### 2.1. Physico and Chemical Characterization of GMFX-o-phdn and Its Complexes

The elemental installation of the separated compounds in addition to their physical properties is shown in Table 1, which are in a decent concurrence with the proposed chemical formulae. The results acquired specified that all the isolated complexes were established by the reaction of metal ions and all the complexes reported here are hydrates with varying degrees of hydration with GMFX-o-phdn in a 1:1 molar ratio for all those metals. All complexes are stable in air, colored, non-hygroscopic powders, and soluble in DMSO and DMF. The molar conductance measurements of all the complexes in DMF solution were in the range of 173.10–272.40 Ω^−1^ mol^−1^ cm^2^ [30] [typified 1:3 electrolytic for (A) complex and 1:2 for (B), (C) and (D) complexes]. The relatively high values signalize electrolytic nature of the complexes which attributed to the non-bonding of the chloride anions to the metal ions in the inner coordination sphere [17,30]. The magnetic susceptibility measurements of the solid complexes were done at room temperature and the calculated values are shown in Table 1. The magnetic moments of Fe(III) and Co(II) complexes were found at 5.81 and 5.10 B.M [31].

### 2.2. IR Spectra and Mode of Bonding

Careful implementing of the IR spectra of complexes and rapprochement with that of the ligand were executed to find out donation information to clarify the technique of bonding of the ligand across diverse metal ions. Thus, a deliberated implementing of the IR spectrum of the ligand was performed and the effectiveness of the metal ion binding in the vibration frequencies was analyzed. The most substantial IR spectral bands of all systems and their assignments are collected in Table 2 and their spectra are offered in Figure S1. The data educe the following observations: the GMFX-o-phdn infrared spectrum reveals the obscurity of bands due to the o-phenylenediamine group ν(NH_2_) and ν(C=O) of gemifloxacin. Instead, newly formed very strong band at 1633 cm^−1^ is obtained showing the complete condensation of the amino groups with the keto group demonstrating the development of the linkage of the Schiff base [32,33].

**Scheme 1:**
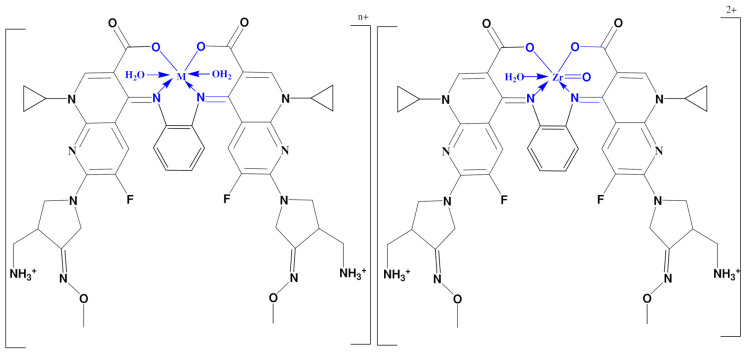
Coordination mode of GMFX–o-phdn with Fe(III), Co(II), Zn(II), and Zr(IV) *n* = 3 for Fe(III) and *n* = 2 for Co(II), Zn(II).

The IR spectra of all complexes containing hydration and coordination water molecules display bands around 3434 cm^−1^ due to ν(O-H) vibration mode of the water molecules which is also supported by elemental analyses [34,35]. Moreover, the appearance of bands around 840, and 600 cm^−1^ in the spectra of all complexes were attributed to rocking and wagging vibration of the coordinated water [36,37]. The two bands spotted at 1715 and 1633 cm^−1^ in the spectrum of GMFX-o-phdn were referred to the stretching vibration of carboxylic ν(COOH) and the azomethine group ν(C=N), respectively [36,37,38]. The missing of the band at 1715 cm^−1^ in all complexes and the shift of the distinctive band of azomethine group to a lower value from 1633 cm^−1^ to 1528 cm^−1^ specified the participation of one oxygen of the carboxylate group and C=N group in the interaction with metal ion forming six-membered rings (Scheme 1) [35]. The bonding is also reinforced by the occurrence of new bands with medium intensity appearing at 640–492 cm^−1^ which assigned to ν(M-O) and ν(M-N) stretching vibrations, respectively [38].

### 2.3. UV–Vis Absorption Spectra

The electronic spectral data are often useful in the estimation of results provided by other methods of structural examination. The assignments of the important electronic spectral bands (Figure S2) of ligand and its metal ion complexes are shown in Table 3. Assorted bands in the electronic spectra of GMFX-o-phdn demonstrated bands at 33,898 and 31,746 cm^−1^ (Table 3) which may be assigned to π-π* and n-π* transitions, respectively; these transitions take place in the condition of unsaturated hydrocarbons containing groups of ketones or groups of azomethine [39,40]. The complexes exhibited new bands in the range of 22,222–20,408 cm^−1^, which may be attributed to a combination of ligand–metal charge transfer (MLCT) [41]. The electronic spectrum of (A) complex shows absorption band at 17,241 cm^−1^ corresponding to ^6^A_1_-^4^T_2_ (G) transitions and the spotted magnetic moment value at 5.81 B.M, suggesting the complex’s high octahedral spin [38,42]. The electronic spectrum of Co(II) complex, [CoC_42_H_58_F_2_N_12_O_13_Cl_2_] (Figure S2), shows recognizable bands at 18,181 and 16,949 cm^−1^; the presence of these bands are consistent with those expected for six coordinate Co(II) complex [43]. These bands can be assigned respectively to ^4^T_1g_ (F) →^4^T_1g_ (P) and^4^T_1g_ (F) →^4^T_1g_ (F) transitions [44,45]. Molar absorptivity (ε) obtained from the electronic spectra of complexes were recorded by using the relation: A = εcl, where A = absorbance, c = 1 × 10^−3^ M, l = length of cell (1 cm) [46].

### 2.4. ^1^H NMR Spectra

The ^1^H-NMR spectra of GMFX-o-phdn, Zn(II) and Zr(IV) compounds were acquired by dissolving them in DMSO-d6 and employing TMS as internal standard Figure S3. The chemical shifts of the diverse types of protons of GMFX-o-phdn, Zn(II) and Zr(IV) compounds were registered in Table 4. ^1^H NMR of Schiff base GMFX-o-phdn (DMSO-d6): δ (1.10–1.34) (1,2)) (m, *J* = 0.72, 4H, -CH_2_ cyclopropane), 1.82 (3) (m, *J* = 5.46, 1H, -CH cyclopropane), 2.32 (-NH) (s, *J* = 6.96, 2H, -NH_2_), 2.50–2.52 (8) (d, *J* = 0.06, 2H, -CH_2_ amine methylene) 3.14- 3.20 (10) (s, 2H, *J* = 0.18, -CH_2_ methylene), 3.82 (s, *J* = 11.46, 2H, H_2_O), 4.37–4.57 (9) (s, *J* = 0.6, 3H, -CH_3_ methyl), 6.90–8.59 (4,5) (s, *J* = 5.07, 2H, H_Ar_) [26]. The signal at 11 ppm (COOH) in the spectrum of the ligand was not registered in Zn(II) and Zr(IV) complexes indicating displacement and complexation of the GMFX-o-phdn with metal ions [41]. Furthermore, the ^1^H NMR spectra for complexes demonstrate a new peak in Zn(II) and Zr(IV) complexes at 3.45 and 3.50 ppm, due to the existence of water molecules in the complexes. All peaks of the GMFX-o-phdn are sitting in spectra of the complexes with some shifts from binding of GMFX-o-phdn to the metal ions [47].

### 2.5. Thermal Studies (TG and DTG)

TG and DTG analyses were performed for affirmation of the molecular structure of the complexes, their thermal stabilities, and revelation of the diverse types of solvents of crystallization. The temperature ranges and weight losses together with DTG peaks are indexed in Table 5 and showed in Figure S4. All synthesized compounds are subjected to thermo gravimetric analysis within temperature range from 25 °C to 1000 °C in nitrogen. The GMFX-o-phdn was thermally decomposed in two sequential decomposition steps. The first and second steps with estimated mass loss of 98.40% (calculated mass loss = 98.65%) within the temperature ranges 32–114 °C and 114–810 °C may be attributed to the loss of 1.5H_2_O and 18C_2_H_2_ + 2HF + 2NH_3_ + 2C_2_N_2_ + CO_2_ + 2NO_2_ + 2N_2_. The TG curve of complex (A) has three degradation steps, the first one has 7.87% weight loss (calculated weight loss = 7.91%) in the range 33 °C to 133 °C, indicating the removal of five hydrated water molecules. The second step with weight loss 37.20% (calc = 37.40%) at maximum temperature 203 °C supporting the removal of two coordinating H_2_O and 15C_2_H_2_ molecules. The third step with two maxima at 317 °C, 435 °C with 42.80% weight loss (calc: 42.42%), corresponding to the removal of 3C_2_H_2_ + 3HCl + 2HF + CO + 2NH_3_ + 1.5H_2_O + 2NO + N_2_ molecules and forming 0.5Fe_2_O_3_ and five carbon as residue. TG thermograms of the three solid complexes (B), (C), and (D) showed three decomposition steps. The first step occurs at 67, 113, 62, and 74 °C maxima temperatures, respectively, with the mass loss of 8.10%, 9.50%, and 7.90% corresponding to the loss of lattice water. The second step occurs at 212, 326, and 172 °C maxima temperatures, respectively, with the weight loss 38.20%, 30.70%, and 23.70% corresponding to the loss of 15C_2_H_2_ + 2H_2_O, 12C_2_H_2_ + 2H_2_O, and 9C_2_H_2_ + 2H_2_O for (B), (C), and (D) complexes. The third step with 436, 394, 366, and 448 °C maxima temperatures, respectively, with a weight loss 41.17%, 47.80%, and 53.40% giving CoO + 5C, ZnO + 4C, and ZrO2 + 4C as final products. The decomposition mechanisms are only based in speculation and thermal analysis without a complementary technique (gas chromatography). The suggested residues confirmed only on the basis of weight loss% calculation.

### 2.6. Differential Thermal Analysis (DTA)

**Figure 1:**
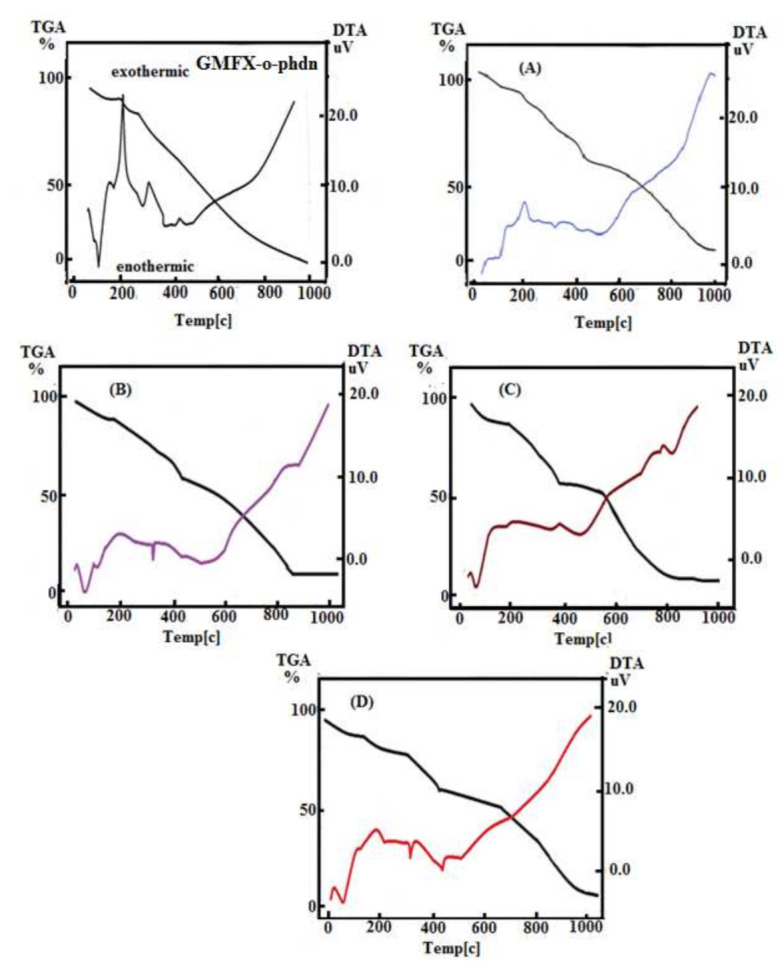
DTA diagram for GMFX-o-phdn and its metal complexes. Where: TGA and DTA are thermo gravimetric and differential thermal analyses, respectively; (**A**) FeC_42_H_58_F_2_N_12_O_13_Cl_3_ complex; (**B**) CoC_42_H_58_F_2_N_12_O_13_Cl_2_ complex; (**C**) ZnC_42_H_60_F_2_N_12_O_14_Cl_2_ complex; and (**D**) ZrC_42_H_56_F_2_N_12_O_13_Cl_2_ complex.

According to the prior DTA survey, the DTA nitrogen thermogram of GMFX-o-phdn with metal complexes under study offers several phases, as shown in Figure 1. In the DTA curves, the chemical modifications that follow the removal of water, anion, and ligand molecules are spotted as exo- or endothermic peaks. At 88, 189, 282, and 439 °C, the GMFX-o-phdn offers these four peaks. The peaks 88, 282, and 439 °C are endothermic peaks with −4.77, −0.35, and −0.55 μV, respectively, except 189 °C is exothermic peak with 11.01 μV with this activation energy. The (A) complex has severalpeaks (endothermic and exothermic) of 1.20, 2.51, and −0.63 μV activation energies at different temperatures of 137, 203, and 328 °C respectively. The complex (B) manifested three peaks at 72, 119, and 329 °C for A. Evaporation of absorbed water is imputed to the first and second peaks and well-defined as endothermic peaks at 72 and 119 °C and activation energy of −3.24 μV and −0.99 μV. At 329 °C, the third peak reflects −1.39 μV of decomposition and activation energy. DTA curve of (C) complex manifests two peaks at 67 and 395 °C. The first endothermic peak with activation energy of −3.84 μV at 67 °C matched to lack of water molecules hydration and the last peak at 395 °C matched to the abstraction of coordinated water and GMFX-o-phdn molecules with activation energy of −0.53 μV. Finally, DTA curve of (D) complex presented four peaks at 80, 197, 331, and 448 °C. The first endothermic peak with activation energy of −4.36 μV at 80 °C matched to lack of water molecules hydration and the last three peaks at 197, 331, and 448 °C matched to the abstraction of coordinated water and Schiff base GMFX-o-phdn molecules with activation energies of 1.50, −1.92, and −1.49 μV.

### 2.7. Calculation of Activation Thermodynamic Parameters

Kinetic parameters of the decomposition steps were specified by non-isothermal approaches such as Horowitz–Metzger (HM) and Coats–Redfern (CR) methods [48,49].

Coats–Redfern equation:

(1) $$\ln\;X=\ln\left[\frac{-\ln\left(1-\alpha \right)}{T^{2}}\right]=\frac{-\mathrm{Ea}}{\mathrm{RT}}+\ln\left[\frac{\mathrm{AR}}{\varphi \mathrm{Ea}}\right]\;\mathrm{for}\;n=1\;.\;$$

(2) $$\ln\;X=\ln\left[\frac{-\ln{\left(1-\alpha \right)}^{1-n}}{T^{2}\left(1-n\right)}\right]=\frac{-\mathrm{Ea}}{\mathrm{RT}}+\ln\left[\frac{\mathrm{AR}}{\varphi \mathrm{Ea}}\right]\;\mathrm{for}\;n\neq 1\;.\;$$

Horowitz–Metzger equation:

(3) $$\ln[-\ln(1-\alpha )]=\frac{E_{a}\theta }{{\mathrm{RT}}_{s}^{2}}\;\mathrm{for}\;n=1\;$$

(4) $$\ln\left[\frac{\left\{1-{\left(1-\alpha \right)}^{1-n}\right\}}{\left(1-n\right)}\right]=\ln(\frac{A}{\Phi }\frac{{\mathrm{RT}}_{s}^{2}}{\mathrm{Ea}})-\frac{E_{a}}{{\mathrm{RT}}_{s}}+\frac{E_{a}\theta }{{\mathrm{RT}}_{s}^{2}}\;\mathrm{for}\;n\neq 1\;$$

The higher activation energy (Ea) values represent the thermal stability of the complexes and are summarized in Table 6. For the subsequent decomposition phases, the increase of the ΔG* value implies that the rate of elimination of the GMFX-o-phdn would be less than that of the prior GMFX-o-phdn and the increase of TΔS* from one phase to another, Figure S5. Compared to the previous complex that needs more energy, TΔS*, this can be due to the structural rigidity of the residual complex following exclusion of one or more GMFX-o-phdn for its rearrangement before undergoing any modulation. Entropy has been shown to have negative numbers in all complexes, which further proposed their stability [50]. The positive value of ΔH* imply that the decay is endothermic.

### 2.8. Antimicrobial Activity

#### 2.8.1. Antifungal Effect

The obtained results showed that the studied GMFX-o-phelin ligand exerted promising antifungal activity against all the tested pathogenic fungi except *M. fructicola* (Table 7). Whereas the highest activity of the parent ligand was observed in the case of *P. italicum* at 1000 ppm (Table 7). On the other hand, all studied metal complexes showed fungicidal effect against *A. flavus* and *B. cinerea* in a dose-dependent manner, where the highest activity was observed against A. flavus using complex (B) at 1000 ppm and against B. cinerea using cycloximide 50 µg/mL, complex (A) 1000 ppm and (B) at 1000 ppm (Table 7). The only antifungal activity against *M. fructicola* was observed in the case of (B) at the three tested concentrations. Regarding *P. italicum*, cycloximide 50 µg/mL showed the highest significant inhibition activity followed by (A) at 1000 ppm, whereas (C) at 1000 ppm showed only moderate activity.

#### 2.8.2. Antibacterial Effect

**Figure 2:**
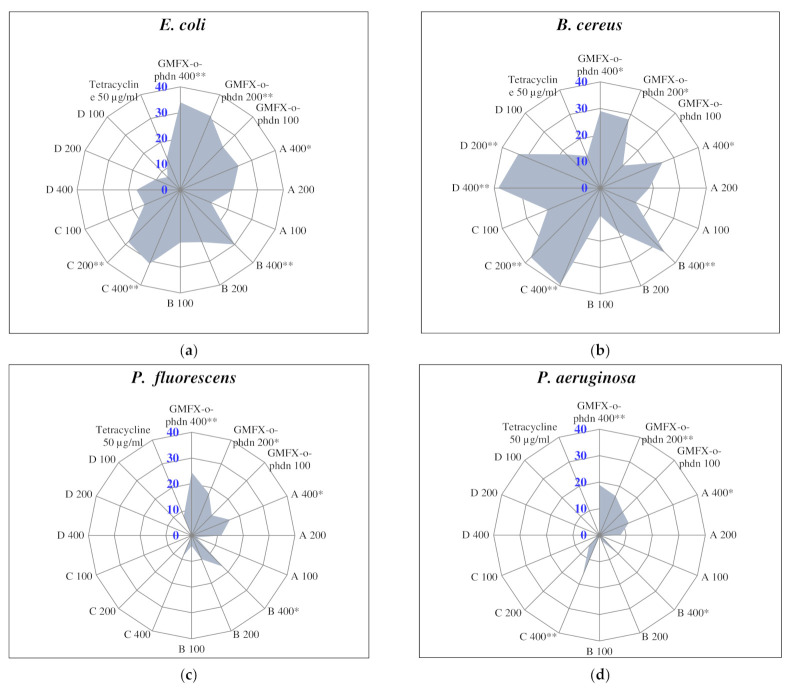
Antibacterial activity assay of GMFX-o-phdn and its metal complexes. Values are recorded as the mean of diameter of inhibition zones (mm) from three replicates ± SDs. (*): statistically significant according to *Tukey* B test at *p* < 0.05: (**): statistically significant at *p* < 0.01.

Regarding the antibacterial activity of the studied GMFX-o-phdn and its metal complexes, the obtained results showed that all tested treatments were able to inhibit the growth of tested pathogenic bacteria especially at the higher tested concentration (400 µg/mL) compared to the positive control tetracycline (50 µg/mL) as illustrated in Figure (2). In particular, the parent ligand showed the highest significant activity against *E.coli*, *P. fluorescens*, and *P. aeruginosa* where the diameters of inhibition zones were measured as 34.0, 24.5, and 19.0 mm, respectively. (C) complex showed the highest significant activity against *E. coli* (31.0 mm), whereas (C) and (D) complexes showed the highest significant activity against *B. cereus* (39.5 and 38.5 mm, respectively) compared to all other treatments (Figure 2).

#### 2.8.3. Mechanism of Antimicrobial Action

The obtained results of antimicrobial test showed that the tested ligands and their metal complexes were able to inhibit the growth of all studied strains in a dose-dependent manner. In particular, the fungicidal effect of the studied compounds could be due to the chemical structure of the free ligand itself as well as the toxicity of the studied metal ions [51,52,53]. The explanation for the promising biological activity of the studied complexes can very well be traced back to several reasons: (i) The concept of cell permeability and chelation process can reduce the polarity of a metal ion through the partial sharing of the positive charge with the donor groups of the ligand; (ii) the chelation process can also increase the delocalization of electrons on the whole chelate ring and enhance the lipophilic nature of the synthesized complex which facilitate its passage into the lipid membranes for penetrating the microbial cells [17,36]; (iii) the potential antimicrobial efficacy of the studied ligand and its metal complexes is thought to be highly related to the specific toxicity of the inorganic salts of metals ions. The obtained results gave a good insight for the possible use of these new prepared compounds for safely controlling different phytopathogens.

On the other hand, the antifungal and antibacterial activity of the studied gemifloxacin ligand and its metal complexes could be correlated also to their ability to inhibit both DNA gyrase and DNA topoisomerase IV enzymes [54]. Furthermore, various metal ions as cobalt, copper, nickel, and zinc were potentially used against several pathogens, where they form low molecular weight complexes and therefore penetrate more efficiently into microbial cells [54].

Recently, most of the antimicrobial drug resistance of some bacterial pathogens is a critical world issue especially for some important pathogens such as: *E. coli*, *P. aeruginosa*, *Enterococcus* spp., and *Staphylococcus* spp. [55,56]. Therefore, search for effective treatments and control of such serious microorganisms remains an important challenge for many researchers all over the world. Several research reported the antimicrobial activity of fluoroquinolones ligands against many pathogenic microorganisms, but they are less active against G-ve bacteria than ciprofloxacin [57,58]. In particular, gemifloxacin ligand demonstrated promising antimicrobial activity, compared to ciprofloxacin and levofloxacin, especially against G +ve bacteria such as: *S. aureus*, penicillin-susceptible and penicillin-resistant strains of *Streptococcus pneumoniae* [59,60,61,62]. On the other hand, some fluoroquinolones ligands such as gemifloxacin and moxifloxacin are less active against *P. aeruginosa* as is ciprofloxacin, hence the obtained results showed promising antibacterial effect against this pathogenic bacterial either in the case of GMFX-o-phdn or the complexes (A), (B), and (C). On the same context, the obtained results of the current research are in agreement with Morissey and Smith [63] who reported that the following three studied quinolones ligands (ofloxacin, levofloxacin, and ciprofloxacin) showed higher antibacterial activity against *P. aeruginosa* than other G-ve bacteria [64]. Meanwhile, the use of many fluoroquinolones such as gatifloxacin, moxifloxacin, and gemifloxacin in clinical field for controlling some pathogenic microorganisms resulted to be safe and had no cytotoxic effects. However, there is still concern about the possible emergence of pathogens resistance [65,66].

### 2.9. Antioxidant Activity

**Figure 3:**
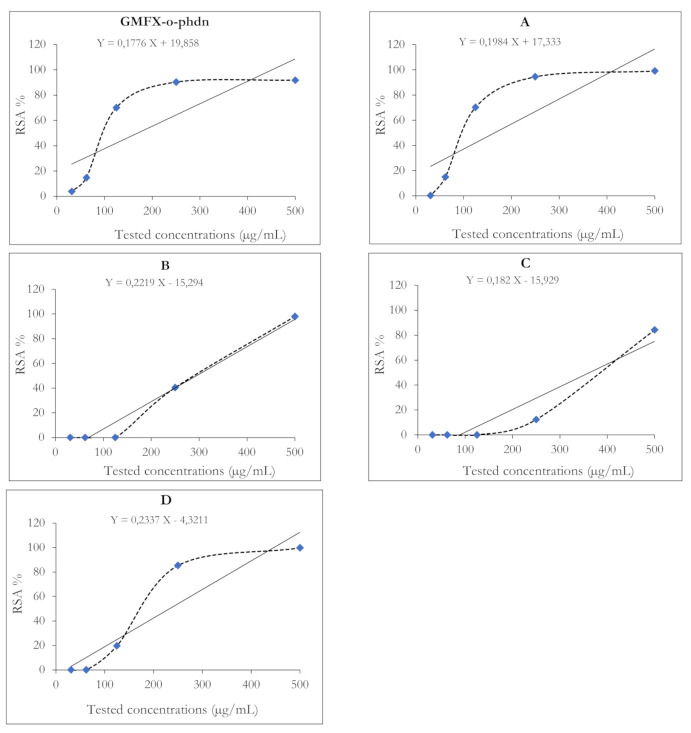
The radical scavenging activity (RSA%) of the studied GMFX-o-phdn and its metal complexes using ABTS method. Where IC_50_ was calculated following the trend line equation equal to: <1, 437, 548, 472, and 673 µg/mL for GMFX-o-phdn, (**A**) Fe, (**B**) Zn, (**C**) Zr, and (**D**) Co, respectively.

Results of the antioxidant activity of GMFX-o-phdn and its metal complexes showed strong antioxidant effect either at 500 or 250 µg/mL (Figure 3). In particular, the parent ligand and G.Fe complex showed greater antioxidant capacity at low tested concentrations than that of other metal complexes where the IC_50_ of GMFX-o-phdn was 169.7 and 164.6 µg/mL, respectively (Table 8). Whereas the highest significant antioxidant activity was reported for the metal complexes observed in the case of (A) complex at 500 µg/mL (Figure 3). On the other hand, (B) and (C) complexes showed the lowest antioxidant activity where the IC_50_ was 300.2 and 362.2 µg/mL, respectively compared to other tested compounds (Table 8). The RSA % of the GMFX-o-phdn metal complex could be due to their hydrogen donating ability [51,67]. In addition, the high scavenging activity of the parent ligand may explain its moderate antimicrobial effect because the reduced compounds are unable to penetrate microbial cell walls, which is characterized by its negative charge, therefore these compounds became difficult to break down and lose the microbe cytoplasm [51].

## 3. Materials and Methods

### 3.1. Materials and Reagents

Whole chemicals applied were of the analytical reagent grade (AR), and of highest purity available. They include gemifloxacin (GMFX), ortho-phenylenediamine (o-phdn), ferric chloride, cobalt chloride hexahydrate, zinc chloride monohydrate, zirconyl chloride heptahydrate, potassium dichromate, concentrated sulfuric acid, commercial grade concentrated nitric acid 69%, hydrogen peroxide 20%, disodium salt EDTA, ammonium hydroxide, ammonium chloride, Variamine Blue, Eriochrome Black T, gallein (Pyrogallolphthalein), and silver nitrate and were provided by Obour Pharmaceutical Industrial Company (Cairo, Egypt), Sigma Aldrich chemicals (Darmstadt, Germany), respectively. Organic solvents used were absolute ethyl alcohol and dimethylformamide (DMF). Distilled water was commonly applied in all preparations. All glassware were steeped overnight in chromic mixture (potassium dichromate + concentrated sulfuric acid) rinsed thoroughly with bidistiled water and dried in an oven at 100 °C.

### 3.2. Preparation of GMFX-o-phdn Schiff Base

**Scheme 2:**
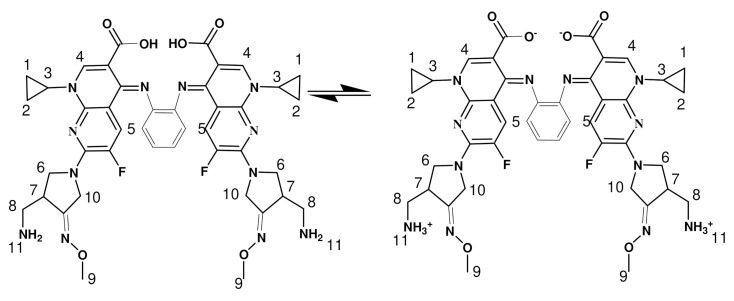
Chemical structure of GMFX-o-phdn.

An ethanolic solution was made by mixing GMFX (2 mmol, 0.77 g) with o-phenylenediamine (1 mmol, 0.108 g) then was once refluxed in the existence of 1 mL of glacial acetic acid for 4 h. The resulting combination was once settled on a water bath then cooled down to 0 °C. The dark red precipitate was filtered off, washed multiple times with ethanol, and dried below vacuum over CaCl_2_ (Scheme 2).

### 3.3. Preparation of Metal Complexes

The brown [Fe(GMFX-o-phdn)(H_2_O)_2_]Cl_3_.5H_2_Ocomplex, (A) was prepared by mixing 0.5 mmol (0.425 g) of GMFX-o-phdn with 30 mL absolute ethanol with 0.5 mmol (0.081 g) of FeCl_3_ in 20 mL ethanol. The mixture was refluxed for 3 h and the precipitate was filtered off and dried under vacuum over anhydrous CaCl_2_. The dark green, dark brown, black solid complexes [Co(GMFX-o-phdn)(H_2_O)_2_]Cl_2_·5H_2_O (B); [Zn(GMFX-o-phdn)(H_2_O)_2_]Cl_2_·6H_2_O (C), and [ZrO(GMFX-o-phdn)(H_2_O)]Cl_2_·5H_2_O (D) were prepared in a similar method described above by using CoCl_2_·6H_2_O, ZnCl_2_·H_2_O and ZrOCl_2_·8H_2_O, respectively.

### 3.4. Instruments

The elemental analyses were performed using a Perkin Elmer 2400 CHN elemental analyzer. The M % content was evaluated using three analytical methods like complexometric titration, thermogravimetry, and atomic absorption. For complexometric titration A digestion procedure was performed to breakdown the metal complexes. Digestion was carried out by addition of 2 mL nitric acid and 1 mL of hydrogen peroxide to the beaker containing a measured weight of metal complex. Then the beaker was placed on the hot plate temperature but not higher than 85 °C and covered with an elevated watch glass and allowed to cool and dilute to 25 mL in a volumetric flask with bi-distilled water. The percentage of the metal ions were determined gravimetrically by transforming the solid products into metal oxide, and also by using atomic absorption method [25]. Atomic absorption analysis was carried out by direct method to estimate the total metal content at the corresponding wavelength. Several reference standard solutions of each metal were prepared with a specific concentration. Spectrometer model PYE-UNICAM SP 1900 fitted with the corresponding lamp was used for this purpose. FT-IR spectra in KBr discs were recorded in the range from 4000–400 cm^−1^ with FT-IR 460 PLUS Spectrophotometer.^1^H NMR spectra were recorded on Varian Mercury VX-300 NMR Spectrometer using DMSO-d_*6*_ as the solvent. TG-DTG measurements were done under N_2_ atmosphere within the temperature range from room temperature to 1000 °C using TGA-50H Shimadzu, the mass of sample was accurately weighted out in an aluminum crucible. Electronic spectra were analyzed using UV-3101PC Shimadzu. The absorption spectra were recorded as solutions in DMSO-d_*6*_. Room temperature magnetic susceptibilities of the powdered samples were analyzed on a Sherwood scientific magnetic balance using Gouy balance at room temperature using Hg[Co(CSN)_4_] as the calibrant. Melting points were recorded on a Buchi apparatus. All measurements were carried out at ambient temperature with freshly prepared solutions. The molar conductance of 1 × 10^−3^ M solutions of the ligands and their complexes in DMF was measured at room temperature using CONSORT K410.

### 3.5. Antifungal Activity Assay

*Tested fungi*. The following tested phytopathogenic fungi, *Monilinia fructicola*, *Aspergillus flavus*, *Penicillium italicum,* and *Botrytis cinerea*, were cultured on potato dextrose agar (PDA) and stored at 4 °C as pure cultures in the mycotheca of School of Agricultural, Forestry, Food and Environmental Sciences (SAFE), University of Basilicata, Potenza, Italy. All tested fungi were previously identified by morphological and molecular methods. *Fungicidal assay*. The fungicidal activity of the tested compounds was determined by incorporation assay in PDA medium [65] at concentrations 1000, 800, and 400 µg/mL. PDA Petri dishes (Ø 90 mm) were inoculated with each single fungal disks (Ø 5 mm). All plates were incubated at 22 ± 2 °C for 96 h and the antifungal effect was evaluated by measuring the diameter of the mycelium growth (mm). The growth inhibition percentage (GI%) was calculated according to Zygadlo et al. [66] (Formula 1) compared to cycloheximide 50 µL/mL.

(5) $$\mathrm{GI}\;\left(\%\right)=\frac{\;\left(\mathrm{Gc}-\mathrm{Gt}\right)}{\mathrm{Gc}}\;\times \;100$$

where GI (%) is the percentage of mycelium growth inhibition, Gc is the average diameter of fungal mycelium in PDA (control), and Gt is the average diameter of fungal mycelium on the treated PDA dish.

### 3.6. Antibacterial Activity Assay

*Tested bacteria.* The tested pathogenic bacterial strains *Escherichia coli*, *Bacillus cereus*, *Pseudomonas fluorescens,* and *P. aeruginosa* were conserved as pure cultures in the collection of SAFE.

*Bactericidal assay*. The antibacterial activity of the tested compounds were evaluated following the disc diffusion method [65,66]. For this trial, the bacterial suspension of each tested strain was prepared in sterile Millipore H_2_O and incorporated with soft agar (0.7%) at ratio 1:9 (*v/v*) to reach 10^8^ colony forming units (CFU/mL). Four milliliter of each suspension was poured in Petri dishes (Ø 90 mm) filled with king B nutrient media (KB). Blank discs (6 mm) (OXOID, Milan, Italy) were placed on each Petri dish and 15 µL of each tested compound was added at 400, 200, and 100 µg/mL concentrations. Tetracycline (50 µg/mL) was used as positive control. The antimicrobial effect was determined after 24 h at 37 °C by measuring the average diameter of inhibition zones (mm) ±SDs of three replicates.

### 3.7. Antioxidant Activity

Antiradical activity of the studied compounds was evaluated using 2,2′-azinobis (3-ethylbenzthiazoline-6-acid) (ABTS^•+^) assay following the methodological procedures of the basic principles of Martysiak-Żurowska and Wenta [68]. The ABTS method is considered the most sensitive method and is characterized by higher repeatability for observing the kinetics of specific enzymes. The detectability and sensitivity of ABTS^•+^ are higher than the DPPH (2,2-diphenyl-1-picrylhydrazyl) assay [51,69]. The stock solution of the ABTS radical was prepared by dissolving 38 mg of ABTS in 10 mL of an aqueous sodium persulphate solution (2.45 mM). The mixture was dark stored for 16 h. For the analysis, 1 mL of stock ABTS^•+^ solution was diluted in ethanol (1:30). Twenty microliter of sample was added to 980 µL of ABTS^•+^ solution for 2 h in dark at room temperature. After centrifuging (5 min, 8000 rpm), absorbance was measured at 734 nm against the reference solvent (ethanol). The solutions were prepared fresh for the analysis and all determinations were carried out in triplicate. Evaluation of the radical scavenging activity (RSA%) of the studied ligand and its metal complexes was carried out at 250, 500, 1000, and 2000 µg/mL concentrations (Formula (2)):

RSA % = (1 − A_t_/A_c_) × 100%; (6)

where A_t_ is the absorbance of sample and A_c_ is the absorbance of colorimetric radical substance without sample.

### 3.8. Statistical Analysis

The obtained results of the antimicrobial assays were subjected to one-way ANOVA for the statistical analysis. The significance level of the outfindings was checked by applying Tukey B Post Hoc multiple comparison test with a probability of *p* < 0.05 using statistical Package for the Social Sciences (SPSS) version 13.0 (Prentice Hall: Chicago, IL, USA, 2004).

## 4. Conclusions

Chemical structures of a novel GMFX-o-phdn Schiff base ligand and its mononuclear metal complexes were portrayed employing diverse physicochemical techniques. The current research disclosed an octahedral geometry around the metal complexes as specified from UV-Visible spectra and magnetic moment measurements. IR spectra disclosed that the bis-Schiff base ligand acts as a tetra dentate ligand and its pattern of coordination is through the nitrogen atoms of the azomethine and oxygen of the carboxylate group. The thermal behavior was studied in order to give an idea about thermal decomposition of the complexes. Moreover, ^1^H NMR spectra for complexes demonstrate a new peak at 3.45 and 3.50 ppm, due to the existence of water molecules in the complexes. On the other hand, the studied complexes exhibited promising antimicrobial activity against most of the tested phytopathogens and strong antioxidant effect.

## Figures and Tables

**Table 1:** Elemental analysis and physico-analytical data for (GMFX-o-phdn) and its metal complexes.

Compounds M.Wt. (M.F.)	Yield %	Mp/°C	Color	Found (Calcd.) (%)					μ_eff_(B.M)	Λ Ω^−1^ mol^−1^ cm^2^
				C	H	N	M	Cl		
(GMFX-o-phdn)1.5H_2_O 877.762 (C_42_H_47_F_2_N_12_O_7.5_)	80.00	190	Dark red	57.29 (57.41)	5.26 (5.35)	19.02 (19.13)				17.60
(A) 1138.957 (FeC_42_H_58_F_2_N_12_O_13_Cl_3_)	82.00	290	Brown	44.11 (44.25)	5.00 (5.09)	14.70 (14.75)	4.78 (4.90)	9.23 (9.33)	5.81	272.40
(B) 1106.592 (CoC_42_H_58_F_2_N_12_O_13_Cl_2_)	84.25	285	Dark green	45.40 (45.54)	5.18 (5.24)	15.10 (15.18)	5.12 (5.32)	6.29 (6.40)	5.10	173.10
(C) 1131.042 (ZnC_42_H_60_F_2_N_12_O_14_Cl_2_)	86.10	270	Dark brown	44.41 (44.56)	5.11 (5.30)	14.76 (14.85)	5.70 (5.78)	6.13 (6.26)		176.20
(D) 1136.886 (ZrC_42_H_56_F_2_N_12_O_13_Cl_2_)	87.15	300	Black	44.26 (44.33)	4.81 (4.92)	14.60 (14.77)	7.96 (8.02)	6.14 (6.23)		175.30

**Table 2:** Selected infrared absorption frequencies (cm^−1^) for GMFX-o-phdn and its metal complexes.

Compounds	ν(O-H); H_2_O; COOH	ν(C=O); COOH	ν_as_(COO^-^)	ν(C=N)	ν_s_(COO^-^)	ν(Zr=O)	ν(M-O), ν(M-N)
GMFX-o-phdn	3427mbr	1715s	-----	1633 s		-	635 w and 548 m
(A)	3436sbr	-	1635vs	1573 m	-----	----	639 w and 492 w
(B)	3434mbr	-	1635vs	1570 m	1391 w	-----	638 m and 500 w
(C)	3432sbr	-	1634vs	1578 s	1387 w	-	637 m and 536 m
(D)	3432sbr	-	1638vs	1528 m	1360 m	813 m	640 m and 497 w

Keys: s = strong, w = weak, m = medium, br = broad, ν = stretching.

**Table 3:** UV-Vis. spectra for GMFX-o-phdn and its metal complexes.

Compounds	Intra Ligand and Charge Transfer	(M^−1^ cm^−1^)	d-d Bands	(M^−1^ cm^−1^)
GMFX-o-phdn	33,898, 31,746	452, 155		
(A)	34,129, 30,769, 22,222	453, 158, 100	17,241	70
(B)	34,246, 31,250, 20,408	453, 155, 80	18,181, 16,949	60, 50
(C)	34,246, 30,769, 21,276	452, 160, 67		
(D)	34,129, 30,769, 20,408	452, 172, 69		

**Table 4:** ^1^H NMR values (ppm) and tentative assignments for GMFX-o-phdn, (C) and (D) metal complexes.

GMFX-o-phdn	(C)	(D)	Assignments
1.10–1.34	1.23–1.30	1.28–1.36	*δ*H, -CH_2_ cyclopropane
1.82	1.61	1.90	*δ*H, -CH cyclopropane
2.32	2.14	2.15	*δ*H, -NH_2_; piperazine
2.50–2.52	2.31–2.90	2.32–2.88	*δ*H, -CH_2_*,* amine methylene
3.14–3.20	3.21	3.19	*δ*H, -CH_2_*,* methylene
3.82	3.45	3.50	*δ*H, H_2_O
4.37–4.57	4.10	4.12–4.22	*δ*H, -CH_3_ methyl
6.90–8.59	7.07–8.50	6.89–8.52	*δ*H, -CH aromatic
11	--		*δ*H, -COOH

**Table 5:** The maximum temperature T_max_ (°C) and weight loss values of the decomposition stages for GMFX-o-phdn and its metal complexes.

Compounds	Decomposition	Temperature Range (°C)	T_max_(°C)	Weight Loss (%)		Lost Species
				Calc.	Found	
(GMFX-o-phdn)1.5H_2_O	First step	32–114	82	3.08	3.10	1.5H_2_O
	Second step	114–1000	188,293,386	95.56	95.30	18C_2_H_2_ + 2HF + 2NH_3_ + 2C_2_N_2_ + CO_2_ + 2NO_2_ + 2N_2_
	Total loss			98.65	98.40	
	Residue			1.36	1.60	C
(A)	First step	33–133	90,118	7.91	7.87	5H_2_O (lattice)
	Second step	133–241	203	37.40	37.20	15C_2_H_2_ + 2H_2_O (coordinated)
	Third step	241–950	317,435	42.42	42.80	3C_2_H_2_ + 3HCl + 2HF + CO + 2NH_3_ + 1.5H_2_O + 2NO + N_2_
	Total loss			87.73	87.87	
	Residue			12.27	12.13	0.5Fe_2_O_3_ + 5C
(B)	First step	32–131	67,113	8.13	8.10	5H_2_O (lattice) 15C_2_H_2_ + 2H_2_O (coordinated) 3C_2_H_2_ + 2HCl + 2HF + CO + 2NO_2_ + 2H_2_ + 5N_2_ CoO + 5C
	Second step	131–256	212	38.49	38.20	
	Third step	256–850	436	41.19	41.17	
	Total loss			87.81	87.47	
	Residue			12.19	12.53	
(C)	First step	32–134	62	9.55	9.50	6H_2_O (lattice) 12C_2_H_2_ + 2H_2_O (coordinated) 6C_2_H_2_ + C_2_N_2_ + 2HF + 2HCl + 2H_2_ + 5N_2_ ZnO + 4C
	Second step	134–331	326	30.77	30.70	
	Third step	331–870	394	48.24	47.80	
	Total loss			88.56	88.00	
	Residue			11.44	12.00	
(D)	First step	32–131	74	7.92	7.90	5H_2_O (lattice) 9C_2_H_2_ + 2H_2_O (coordinated) 10C_2_H_2_ + 2HCl + 2HF + 2NO_2_ + 5N_2_ ZrO_2_ + 4C
	Second step	131–273	172	23.75	23.70	
	Third step	273–970	366,448	53.27	53.40	
	Total loss			84.94	85.00	
	Residue			15.06	15.00	

**Table 6:** Thermal behavior and Kinetic parameters determined using Coats–Redfern (CR) and Horowitz–Metzger (HM) operated for GMFX-o-phdn and its metal complexes.

Compounds	Decomposition Range (K)	T_s_ (K)	Method	Parameters					R ^a^	SD ^b^
				Ea (KJ/mol)	A (s^−1^)	ΔS* (KJ/mol.K)	ΔH* (KJ/mol)	ΔG* (KJ/mol)		
GMFX-o-phdn	305–387	355	CR	74.28	2.88 × 10^8^	−0.0101	71.33	74.94	0.978	0.155
			HM	84.18	3.25 × 10^10^	−0.0450	81.23	97.24	0.974	0.168
	387–506	387	CR	98.54	1.54 × 10^10^	−0.0520	98.98	119.10	0.978	0.157
			HM	99.61	3.70 × 10^11^	−0.0256	96.39	106.30	0.974	0.168
	496–635	566	CR	125.77	5.88 × 10^9^	−0.0632	121.06	121.07	0.960	0.201
			HM	154.00	1.56 × 10^12^	−0.0167	149.29	158.79	0.962	0.215
	635–1273	659	CR	107.79	4.00 × 10^5^	−0.0173	102.31	113.75	0.963	0.262
			HM	101.99	5.70 × 10^5^	−0.1413	96.52	189.64	0.953	0.297
(A)	306–406	363	CR	26.71	8.321	−0.2289	23.69	106.78	0.998	0.0137
			HM	30.80	0.0046	−0.2911	27.78	133.48	0.997	0.0181
	406–514	391	CR	21.92	0.7427	−0.2496	18.67	116.27	0.999	0.0038
			HM	22.85	0.0029	−0.2955	19.60	135.14	0.999	0.0064
	514–637	476	CR	29.57	1.1254	−0.2478	25.61	143.57	0.999	0.0016
			HM	25.97	1.6232	−0.2447	22.01	138.52	0.999	0.0031
	514–637	590	CR	32.43	0.8072	−0.2523	27.53	176.42	0.999	0.0010
			HM	32.18	1.3047	−0.2483	27.27	173.81	0.999	0.0041
(B)	305–404	340	CR	72.07	2.03 × 10^8^	−0.0869	69.24	98.80	0.978	0.1553
			HM	77.22	9.76 × 10^9^	−0.0547	74.39	93.01	0.974	0.1682
(C)	305–407	335	CR	72.07	2.10 × 10^8^	−0.0865	69.29	98.28	0.978	0.1553
			HM	74.96	6.53 × 10^9^	−0.0579	72.18	91.60	0.974	0.1682
	439–604	599	CR	82.88	1.08 × 10^7^	−0.1159	77.90	147.37	0.969	0.1984
			HM	159.41	1.17 × 10^11^	−0.0387	154.43	177.66	0.965	0.2023
	604–738	667	CR	197.33	7.78 × 10^13^	0.01433	191.78	182.22	0.969	0.1918
			HM	236.79	3.72 × 10^16^	0.06563	231.25	187.47	0.966	0.2020
(D)	305–404	374	CR	72.07	1.95 × 10^8^	−0.0874	69.19	99.53	0.978	0.1553
			HM	80.43	1.71 × 10^10^	−0.0525	77.55	94.99	0.974	0.1682
	694–1272	721	CR	112.49	8.51 × 10^4^	−0.1578	106.50	220.32	0.965	0.2720
			HM	98.62	5.29 × 10^4^	−0.16181	92.63	209.30	0.953	0.3123

Where: (^a^) correlation coefficients *of Arrhenius plots;* (^b^) *standard deviation*; (Ea) *the activation energy;* (ΔS^*^) the entropy of activation; (ΔH*) the enthalpy of activation; and (ΔG*) Gibbs free energy.

**Table 7:** Antifungal activity assay of GMFX-o-phdn ligand and its metal complexes.

Treatment (µg/mL)		Mycelium Growth Inhibition (%)			
		*M. fructicola*	*A. flavus*	*P. italicum*	*B. cinerea*
GMFX-o-phdn	1000	0.0 ± 0.0c	16.1 ± 1.9b	38.9 ± 3.8c	20.0 ± 2.6b
	800	0.0 ± 0.0c	0.0 ± 0.0d	0.0 ± 0.0d	11.1 ± 2.6c
	400	0.0 ± 0.0c	0.0 ± 0.0d	0.0 ± 0.0d	0.0 ± 0.0d
(A)	1000	0.0 ± 0.0c	22.7 ± 4.6b	52.9 ± 4.1b	43.9 ± 4.0a
	800	0.0 ± 0.0c	6.7 ± 2.6c	0.0 ± 0.0d	18.7 ± 2.3b
	400	0.0 ± 0.0c	0.0 ± 0.0	0.0 ± 0.0d	6.7 ± 2.3c
(B)	1000	32.6 ± 4.5c	44.4 ± 5.1a	0.0 ± 0.0d	38.3 ± 5.0a
	800	20.6 ± 2.2b	18.9 ± 6.4b	0.0 ± 0.0d	18.1 ± 2.2b
	400	5.9 ± 1.0c	4.3 ± 1.2d	0.0 ± 0.0d	7.9 ± 2.2c
(C)	1000	0.0 ± 0.0c	15.8 ± 2.3b	42.1 ± 1.7c	23.2 ± 3.0b
	800	0.0 ± 0.0c	8.9 ± 2.6c	0.0 ± 0.0d	0.0 ± 0.0d
	400	0.0 ± 0.0c	0.0 ± 0.0d	0.0 ± 0.0d	0.0 ± 0.0d
(D)	1000	0.0 ± 0.0c	16.7 ± 3.8b	0.0 ± 0.0d	21.8 ± 1.5b
	800	0.0 ± 0.0c	7.8 ± 1.3c	0.0 ± 0.0d	10.4 ± 1.5c
	400	0.0 ± 0.0c	2.6 ± 0.4d	0.0 ± 0.0d	2.4 ± 0.3d
Cycloximide	50 µg/mL	16.1 ± 2.8b	9.7 ± 3.0c	71.1 ± 5.1a	42.2 ± 2.6a

Values were recorded as the mean of growth inhibition percentage of tested pathogenic fungi (three replicates) ± SDs. Values followed by different letters in each vertical column were significantly different according to *Tukey* B test at *p* < 0.05. Cycloheximide: the positive control at 50 µL/mL.

**Table 8:** The absorbance and IC_50_ of antioxidant activity for the studied compounds.

Compound	Absorbance (734 nm) at Different Concentrations (µg/mL)					IC_50_ (µg/mL)
	C.500	C.250	C.125	C.62	C.31	
GMFX-o-phdn	0.160	0.191	0.589	1.685	1.900	169.7
A	0.019	0.110	0.589	1.685	1.974	164.6
B	0.313	1.733	2.326	2.377	2.226	300.2 *
C	0.006	0.290	1.590	2.415	2.196	362.2 *
D	0.040	1.179	2.191	2.214	2.158	232.4

Where: (GMFX-o-phdn): C_42_H_44_F_2_N_12_O_6_; (A): FeC_42_H_58_F_2_N_12_O_13_Cl_3_; (B) CoC_42_H_58_F_2_N_12_O_13_Cl_2_; (C) ZnC_42_H_60_F_2_N_12_O_14_Cl_2_; (D) ZrC_42_H_56_F_2_N_12_O_13_Cl_2_. (*) is the highest IC_50_ of Co and Zn complexes indicating their lower antioxidant activity.

## Data Availability

Not applicable.

## Supplementary Materials

The following are available online at https://www.mdpi.com/article/10.3390/ijms23042110/s1.
