# Supplementary material for: Biochemical Characterization of New Gemifloxacin Schiff Base (GMFX-o-phdn) Metal Complexes and Evaluation of Their Antimicrobial Activity against Some Phyto- or Human Pathogens
Source: Int J Mol Sci. 2022 Feb 14;23(4):2110. doi: 10.3390/ijms23042110 (PMC8877648; doi:10.3390/ijms23042110)
Supplement: Supplementary file 1 [file ijms-23-02110-s001.zip › ijms-1535365-supplementary.pdf]

Supplementary Materials

Biochemical Characterization of New Gemifloxacin Schiff Base (GMFX-o-phdn) Metal Complexes and Evaluation of Their Antimicrobial Activity against Some Phyto- or Human Pathogens

Hazem S. Elshafie[1], Sadeek A. Sadeek[2], Ippolito Camele1, and Amira A. Mohamed[3]

[1] School of Agricultural, Forestry, Food and Environmental Sciences, University of Basilicata, 85100 Potenza, Italy; hs.elshafie@gmail.com

[2] Department of Chemistry, Faculty of Science, Zagazig University, Zagazig 44519, Egypt; s_sadeek@zu.edu.eg

[3] Department of Basic Science, Zagazig Higher Institute of Engineering and Technology, Zagazig 44519, Egypt; amira222283@yahoo.com

* Correspondence: ippolito.camele@unibas.it; Tel.: +39-0971-205544; Fax: +39-0971-205503

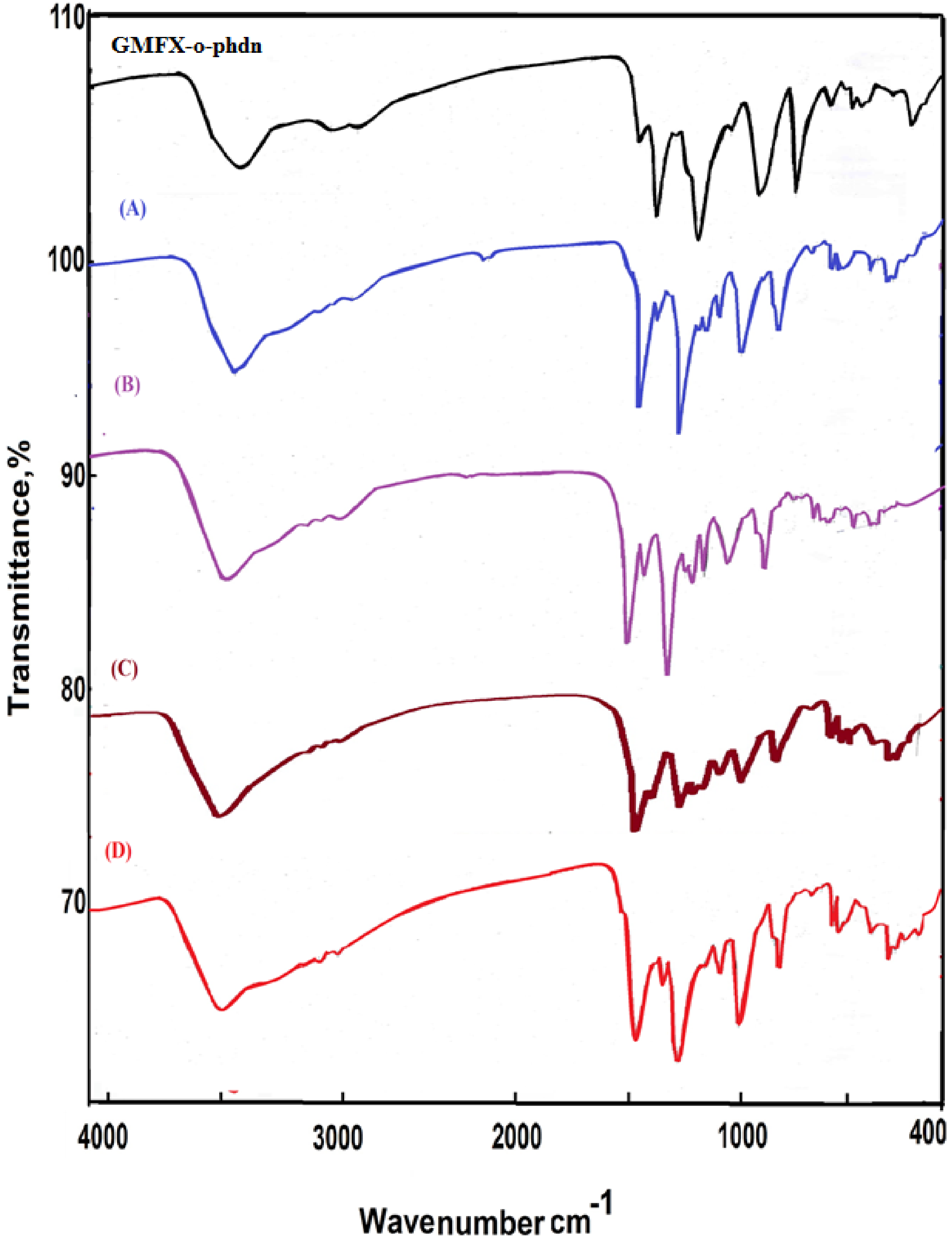

Figure S1. Infrared spectra for GMFX-o-phdn and its metal complexes

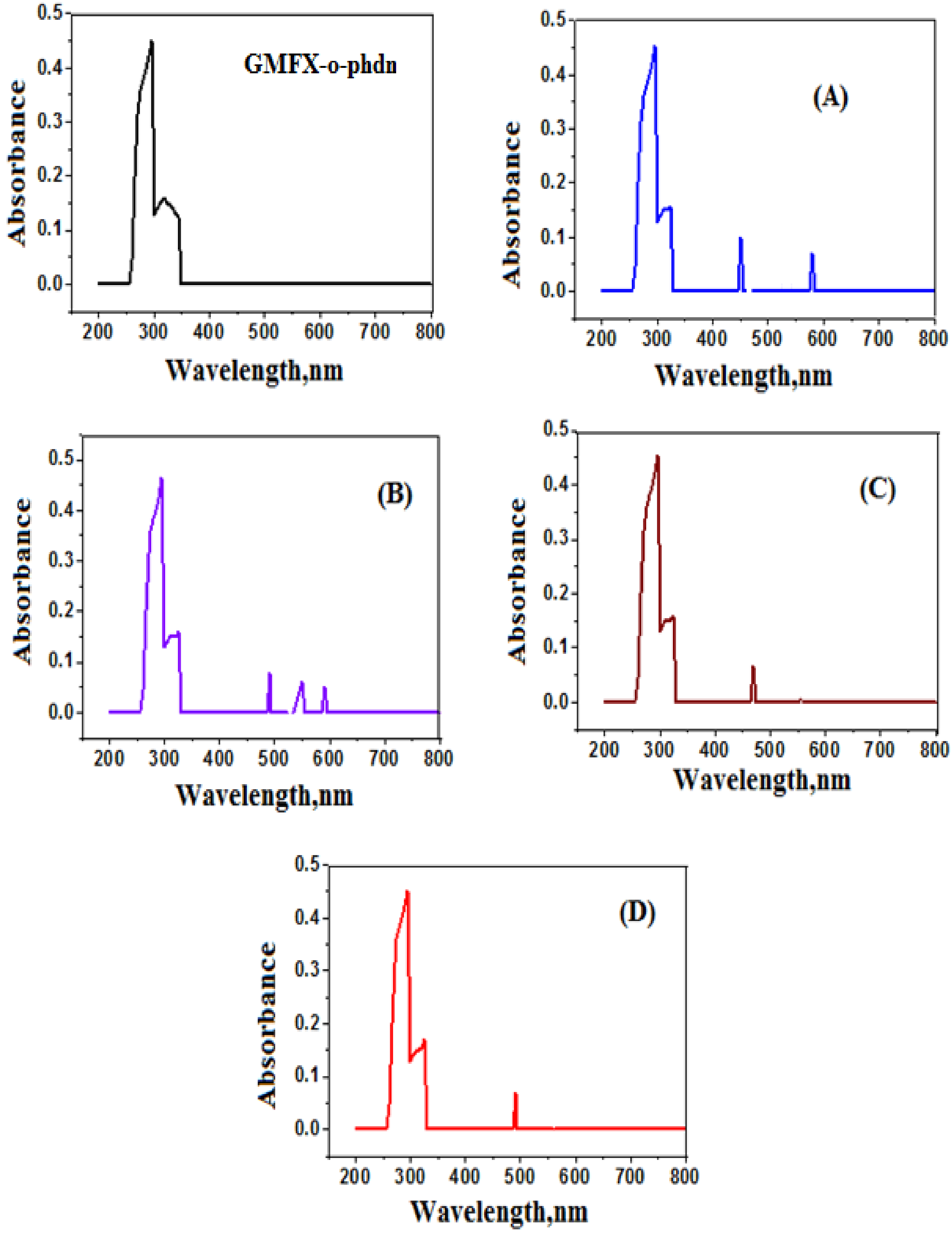

Figure S2. Electronic absorption spectra for GMFX-o-phdn and its metal complexes

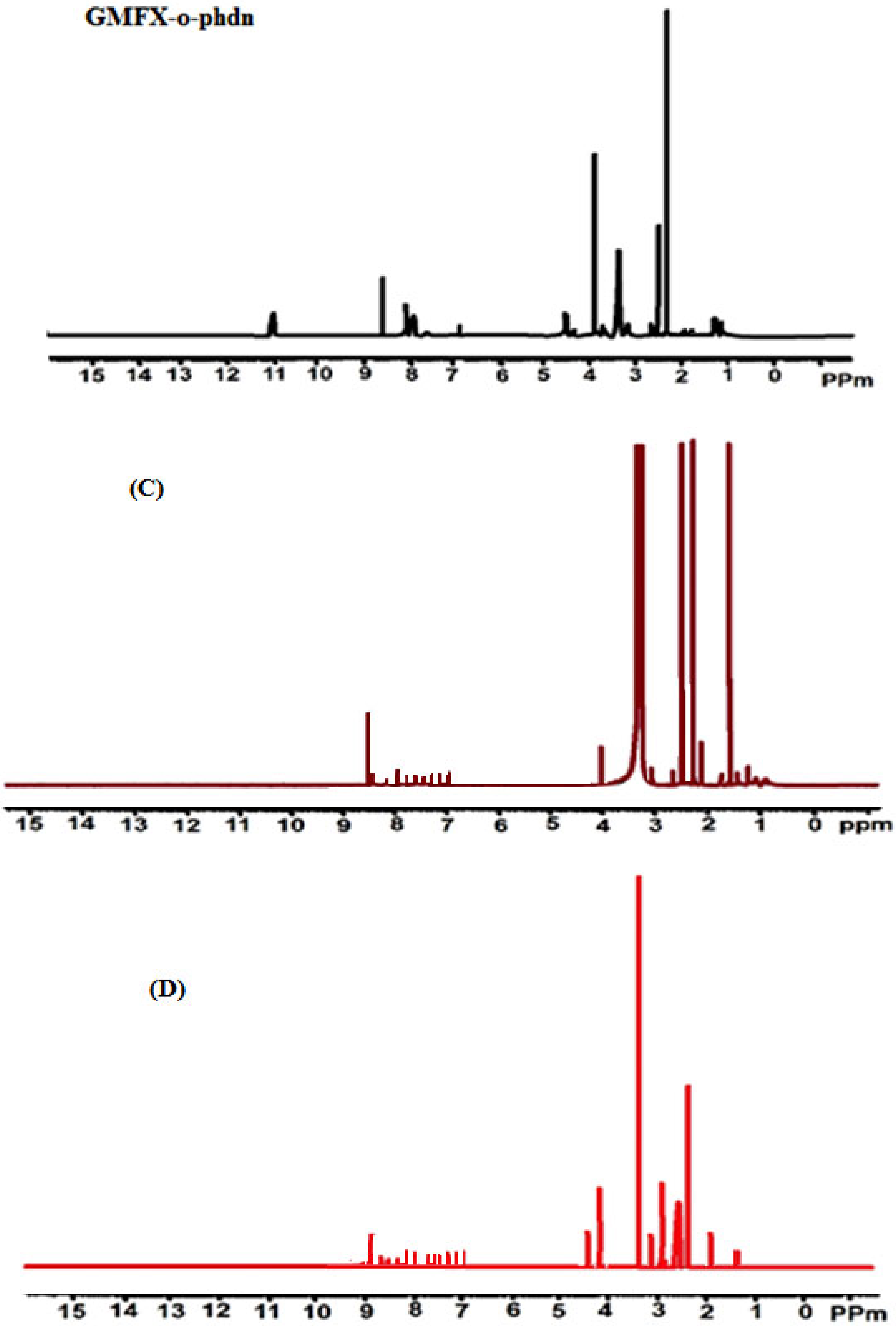

Figure S3. ^{1}H NMR spectra for GMFX-o-phdn, (C) and (D) complexes

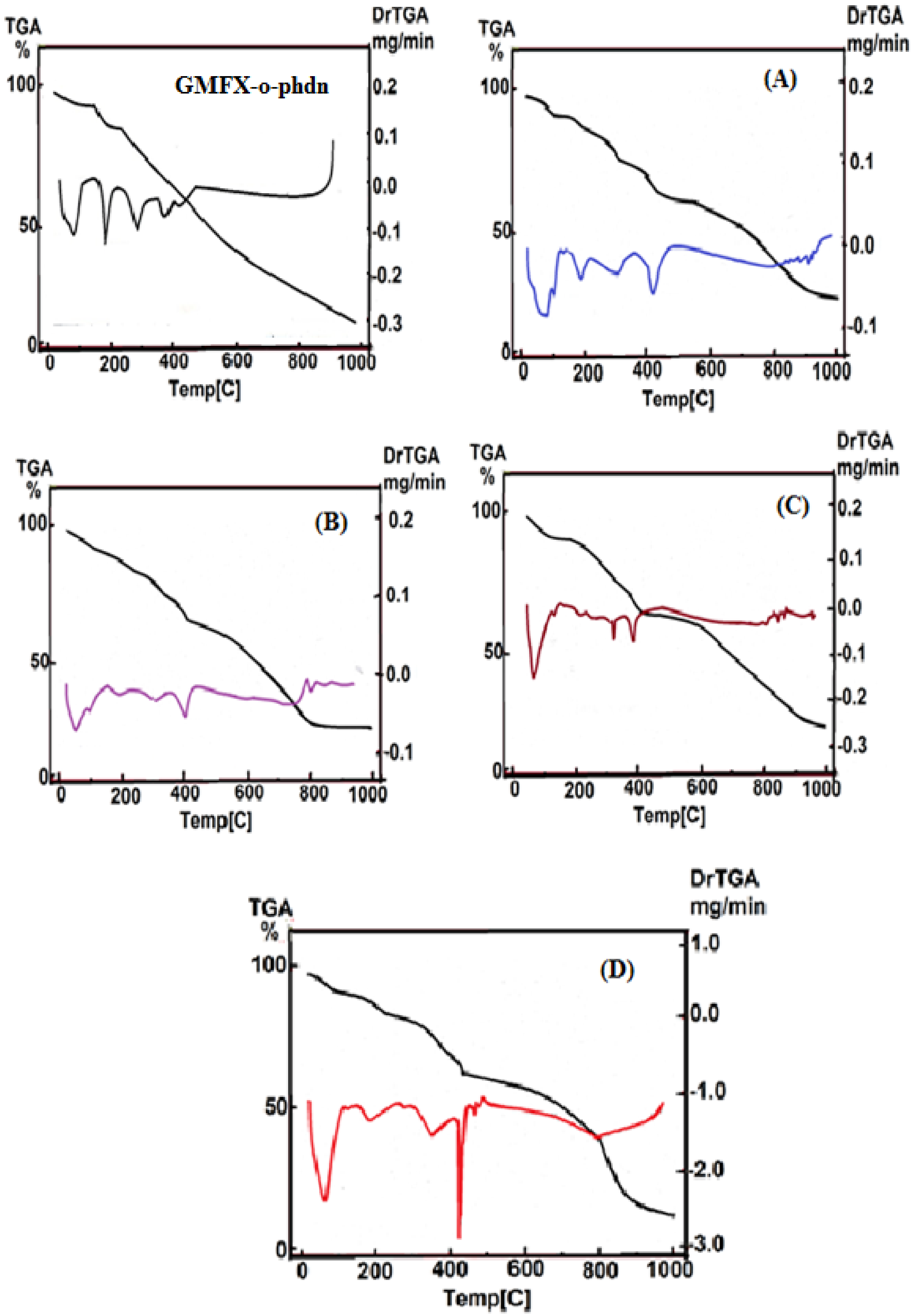

Figure S4. TGA and DTG diagram for GMFX-o-phdn and its metal complexes

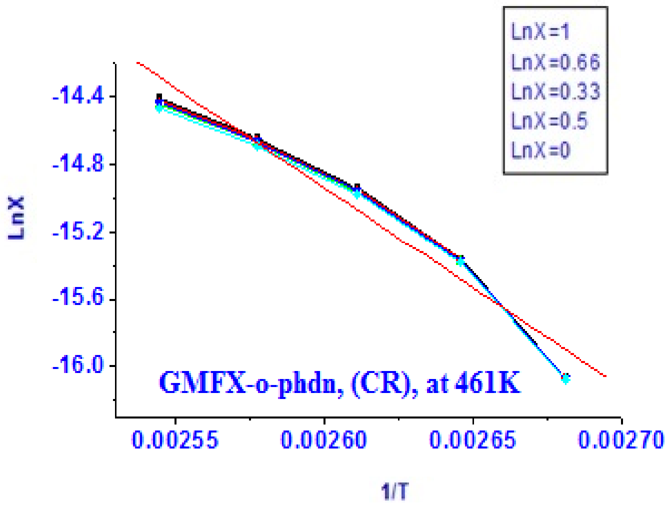
LnX=1
LnX=0.66
LnX=0.33
LnX=0.5
LnX=0
LnX
1/T
GMFX-o-phdn, (CR), at 461K

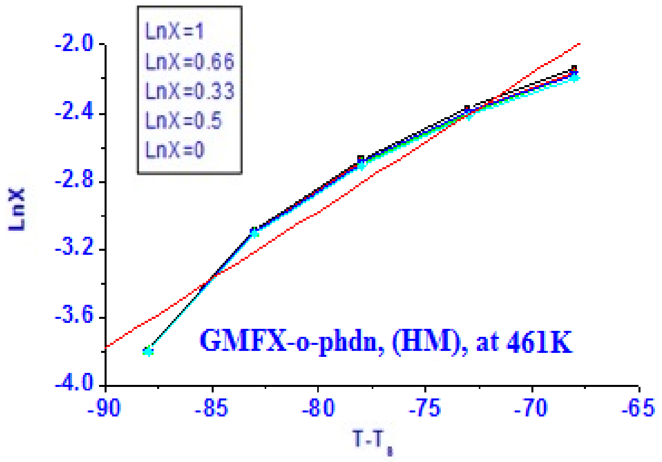
LnX=1
LnX=0.66
LnX=0.33
LnX=0.5
LnX=0
LnX
GMFX-o-phdn, (HM), at 461K

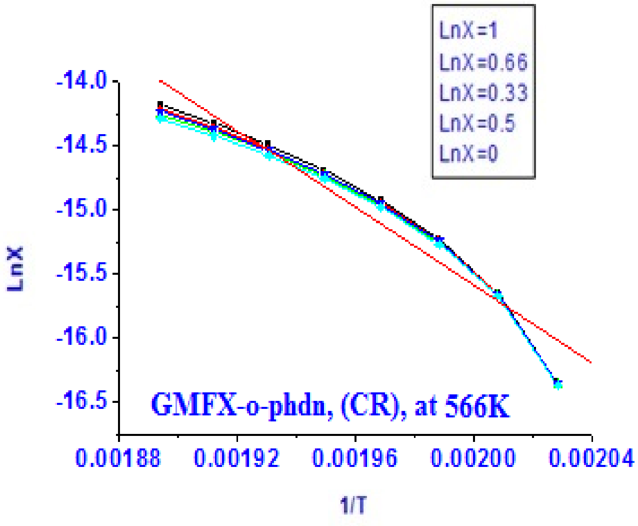
LnX=1
LnX=0.66
LnX=0.33
LnX=0.5
LnX=0
LnX
1/T
GMFX-o-phdn, (CR), at 566K

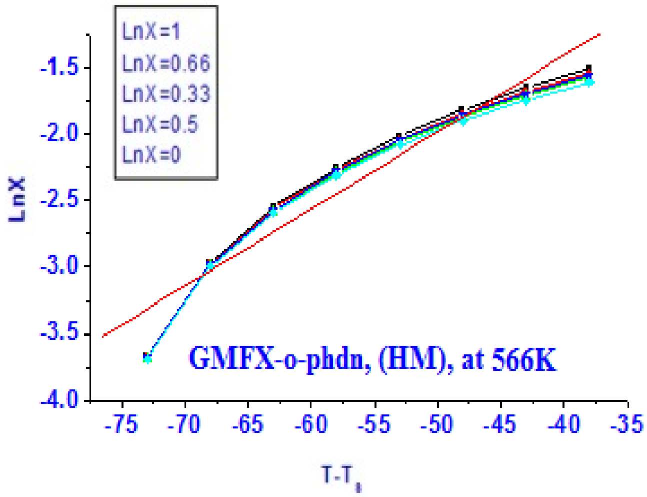
LnX=1
LnX=0.66
LnX=0.33
LnX=0.5
LnX=0
LnX
GMFX-o-phdn, (HM), at 566K

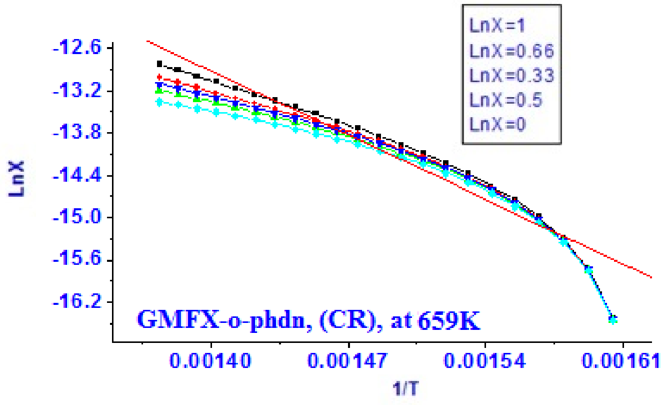
LnX=1
LnX=0.66
LnX=0.33
LnX=0.5
LnX=0
LnX
1/T
GMFX-o-phdn, (CR), at 659K

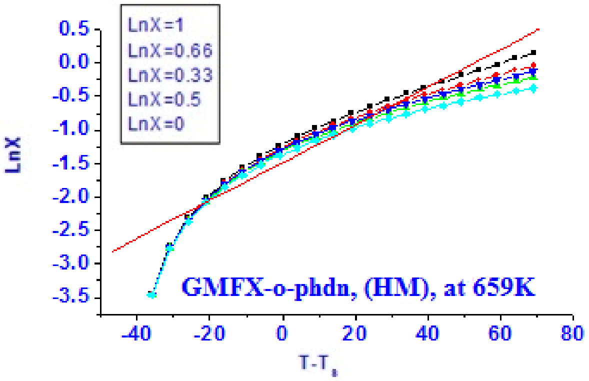
LnX=1
LnX=0.66
LnX=0.33
LnX=0.5
LnX=0
LnX
GMFX-o-phdn, (HM), at 659K

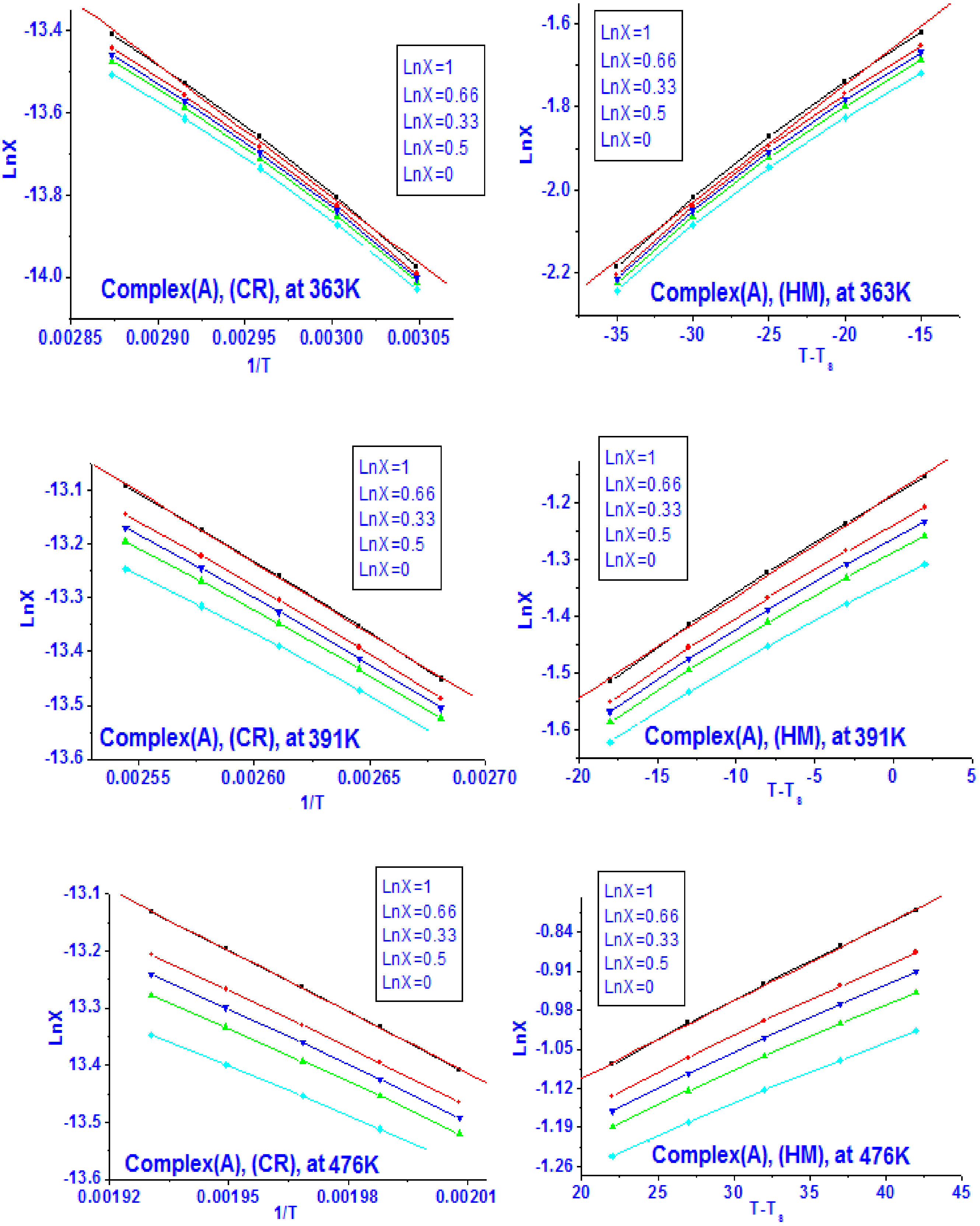
Complex(A), (CR), at 363K
LnX
1/T
LnX=1
LnX=0.66
LnX=0.33
LnX=0.5
LnX=0
Complex(A), (HM), at 363K
T-Ts
Complex(A), (CR), at 391K
Complex(A), (HM), at 391K
Complex(A), (CR), at 476K
Complex(A), (HM), at 476K

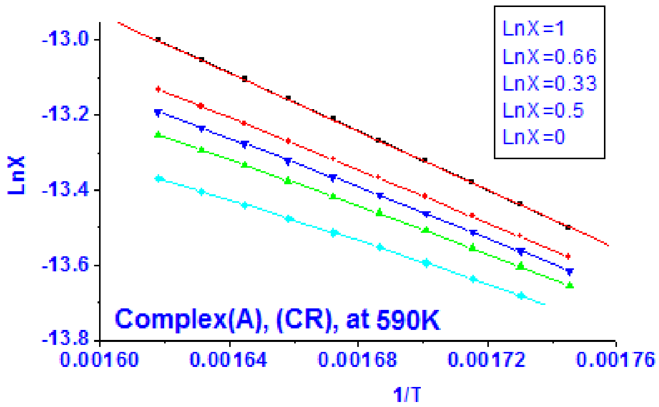
LnX=1
LnX=0.66
LnX=0.33
LnX=0.5
LnX=0
Complex(A), (CR), at 590K
LnX
1/T
-13.0
-13.2
-13.4
-13.6
-13.8
0.00160
0.00164
0.00168
0.00172
0.00176

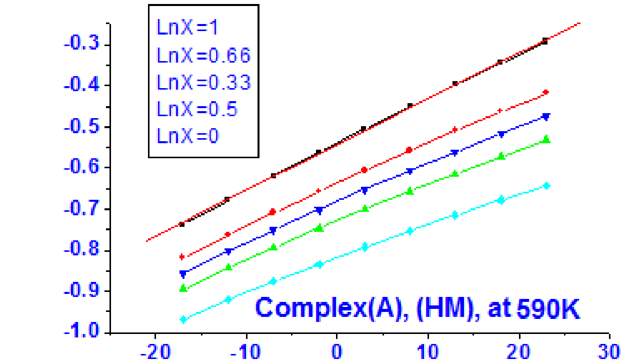
LnX=1
LnX=0.66
LnX=0.33
LnX=0.5
LnX=0
Complex(A), (HM), at 590K
-0.3
-0.4
-0.5
-0.6
-0.7
-0.8
-0.9
-1.0
-20
-10
0
10
20
30

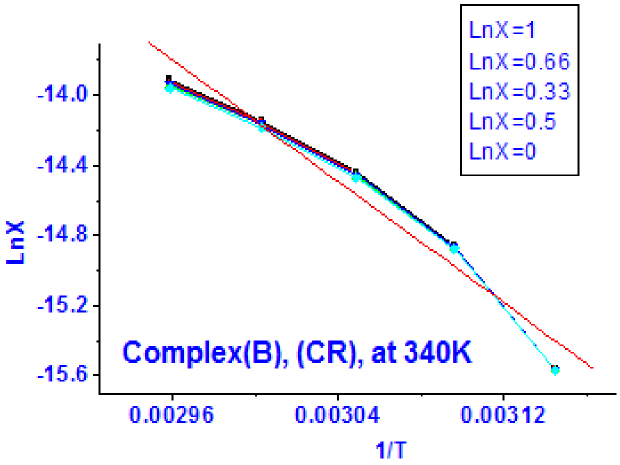
LnX=1
LnX=0.66
LnX=0.33
LnX=0.5
LnX=0
Complex(B), (CR), at 340K
LnX
1/T
-14.0
-14.4
-14.8
-15.2
-15.6
0.00296
0.00304
0.00312

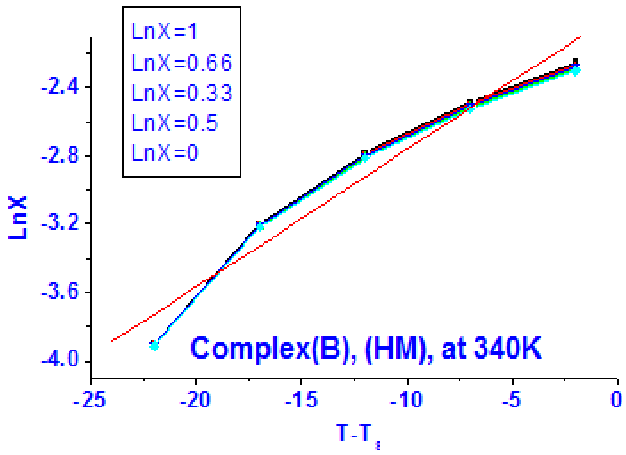
LnX=1
LnX=0.66
LnX=0.33
LnX=0.5
LnX=0
Complex(B), (HM), at 340K
LnX
T-Tg
-2.4
-2.8
-3.2
-3.6
-4.0
-25
-20
-15
-10
-5
0

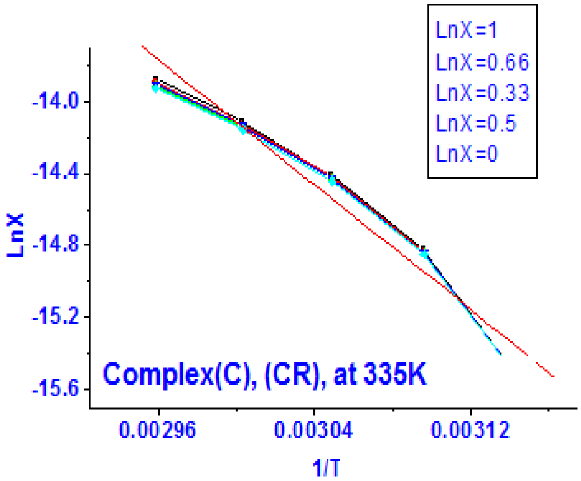
LnX=1
LnX=0.66
LnX=0.33
LnX=0.5
LnX=0
Complex(C), (CR), at 335K
LnX
1/T
-14.0
-14.4
-14.8
-15.2
-15.6
0.00296
0.00304
0.00312

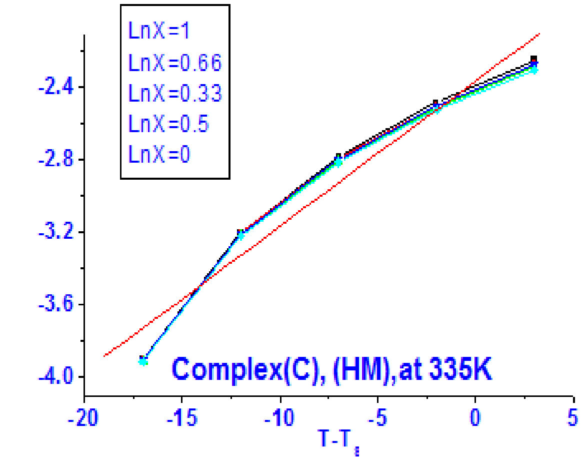
LnX=1
LnX=0.66
LnX=0.33
LnX=0.5
LnX=0
Complex(C), (HM),at 335K
T-Tg
-2.4
-2.8
-3.2
-3.6
-4.0
-20
-15
-10
-5
0
5

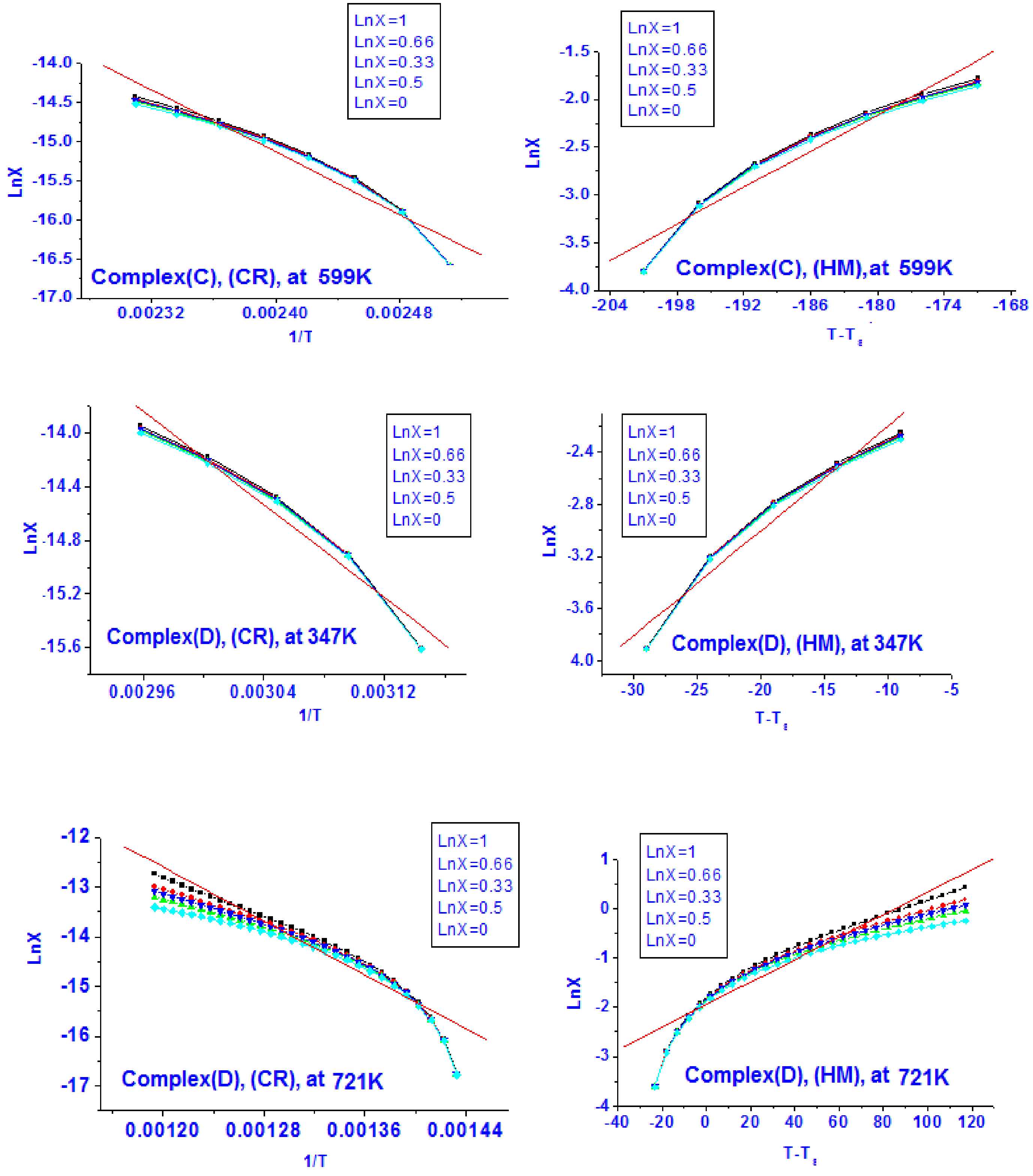

Figure S5. The diagrams of kinetic parameters of GMFX-o-phdn and its metal complexes using Coats-Redfern(CR) and Horowitz-Metzger (HM) equations
